# Acupuncture for the postcholecystectomy syndrome: Protocol for a systematic review and meta-analysis

**DOI:** 10.1097/MD.0000000000016769

**Published:** 2019-08-09

**Authors:** Zihan Yin, Ying Cheng, Qiwei Xiao, Guixing Xu, Han Yang, Jun Zhou, Yanan Fu, Jiao Chen, Ling Zhao, Fanrong Liang

**Affiliations:** School of Acu-Mox and Tuina, Chengdu University of Traditional Chinese Medicine, Chengdu, Sichuan Province, China.

**Keywords:** acupuncture, postcholecystectomy syndrome, protocol, systematic review

## Abstract

**Background:**

Postcholecystectomy syndrome (PCS) is a term used to describe the persistence of biliary colic or right upper quadrant abdominal pain with a variety of postoperative gastrointestinal symptoms. Acupuncture and related treatments have shown clinical effects for PCS in many studies. But the systematic reviews and meta-analyses for them are lacking. We aim to evaluate the efficacy and safety of acupuncture on the treatment of PCS.

**Methods:**

We will search 8 electronic databases, including the Web of Science, PubMed, Cochrane Library, Embase, and 4 Chinese databases (CBM, Wanfang, VIP, and CNKI databases), and additional sources (WHO ICTRP, ChiCTR, Clinical Trials, Grey Literature Database), for potentially eligible studies. Literature retrieval, screening, and data extraction will be conducted by 2 researchers independently. In case of disagreement, a 3rd party shall be consulted to assist judgment. We will use RevmanV.5.3 to perform a fixed effect meta-analysis on the data of clinical homogeneity studies, and evidence's level will be assessed through the method for GRADE.

**Results:**

This systematic review and meta-analysis will put a high-quality synthesis of the efficacy and safety of acupuncture treatment in PCS.

**Conclusion:**

The conclusion of this systematic review will provide evidence to assess acupuncture therapy is an efficacy and safe intervention to treat and control PCS.

**Ethics and dissemination:**

Since this article does not involve patients’ private data, no ethical approval is required. The agreement will be disseminated by peer-reviewed journals or conference reports.

**Trial registration number:**

PROSPERO CRD4201929287.

## 1 Introduction

Cholecystectomy, approximately 700,000 cases performed each year,^[1]^ is the most common gastrointestinal operation performed in the United States. Despite being the most commonly performed operations, sometimes cholecystectomy fails to relieve symptoms.^[2]^ And 10% of patients may develop postcholecystectomy syndrome (PCS) weeks to month's later. PCS was 1st described in 1947 by Womack and Crider.^[3]^ And it is defined as the recurrence of symptoms similar to those experienced before the cholecystectomy.^[4]^ In 5% to 40% of patients with cholelithiasis, regardless of the type of surgery, the symptoms persist postoperatively.^[5,6]^ And the incidence rate of the PCS in this study's population was 19.8%.^[7]^ Approximately 5% to 40% of patients who have undergone cholecystectomy continue to have symptoms of abdominal pain, vomiting, gastrointestinal symptoms (dyspepsia, loose stool, and the like) and are thought to suffer from PCS.^[7–11]^

Causes of PCS are complicated and many can be attributed to extra-biliary causes.^[7]^ So the treatments for PCS include physical therapy, medicine,^[12–14]^ and surgical treatment.^[15]^ In eastern, physical treatment such as acupuncture has a good effect for PCS.^[16–19]^ Acupuncture has been widely used in treating the symptoms.^[20]^ However, to our knowledge, the randomized controlled trials (RCTs) examining the effectiveness and safety of acupuncture for PCS have never been systematically evaluated. Therefore, we will carry out this study to evaluate the evidence of RCTs for acupuncture treatment of PCS.

## 2 Methods

### 2.1 Design and registration of the review

This SR has been registered on PROSPERO and registration number is CRD42019129287 and the protocol is based on the PRISMA-P guidelines.^[21]^

### 2.2 Inclusion criteria for study selection

#### 2.2.1 Type of study

All the studies of acupuncture in the treatment of PCS and the included studies will be all RCTs without limitation on language or publication types restriction. Nonrandomized clinical studies, quasi-RCTs, cluster RCTs, and case reports will be excluded.

#### 2.2.2 Types of participants

Trials involving patients who are diagnosed PCS will be included.

#### 2.2.3 Types of interventions

Acupuncture and related treatments will be used in the intervention group. Studies using acupuncture in experimental group will be included regardless of the treatment length and frequency. And control group will consist of drugs, placebo, sham acupuncture, etc.

#### 2.2.4 Types of outcome measures

##### 2.2.4.1 Primary outcomes

Pain intensity (relevant confirmed pain measurement scales such as visual analog scale), the nausea incidence and the vomiting incidence will be analyzed.

##### 2.2.4.2 Secondary outcomes

1. The recovery of gastrointestinal function: first defecation time, 1st flatus time, 1st bowel sounds time, duration of abdominal distension, etc.
2. Adverse effects (relevant symptoms caused by acupuncture).

### 2.3 Data sources

We will search 8 electronic databases and additional sources, including the Web of Science, PubMed, Cochrane Library, Embase, CBM, Wanfang, VIP, CNKI, and WHO ICTRP, ChiCTR, Clinical Trials, Grey Literature Database, for potentially eligible studies. RCTs on acupuncture treatment in patients with PCS will be searched for independently by 2 reviewers in those sources.

### 2.4 Search strategy

The details are adjusted according to the specific sources including CBM, CNKI, WF, VIP, Web of Science, Embase, PubMed, Cochrane Library, WHO ICTRP, ChiCTR, Clinical Trials, and Grey Literature Database. The search strategy for PubMed is shown in Table 1.

**Table 1:**
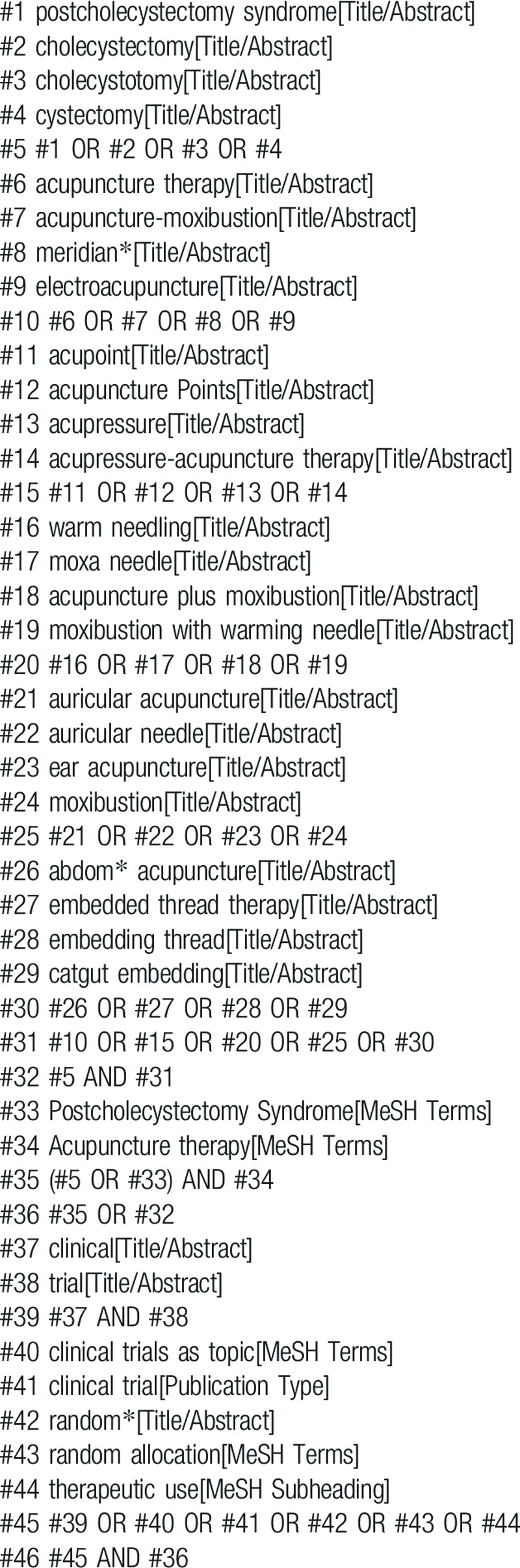
Search strategy for the PubMed database.

### 2.5 Data collection and analysis

#### 2.5.1 Selection of studies

**Figure 1:**
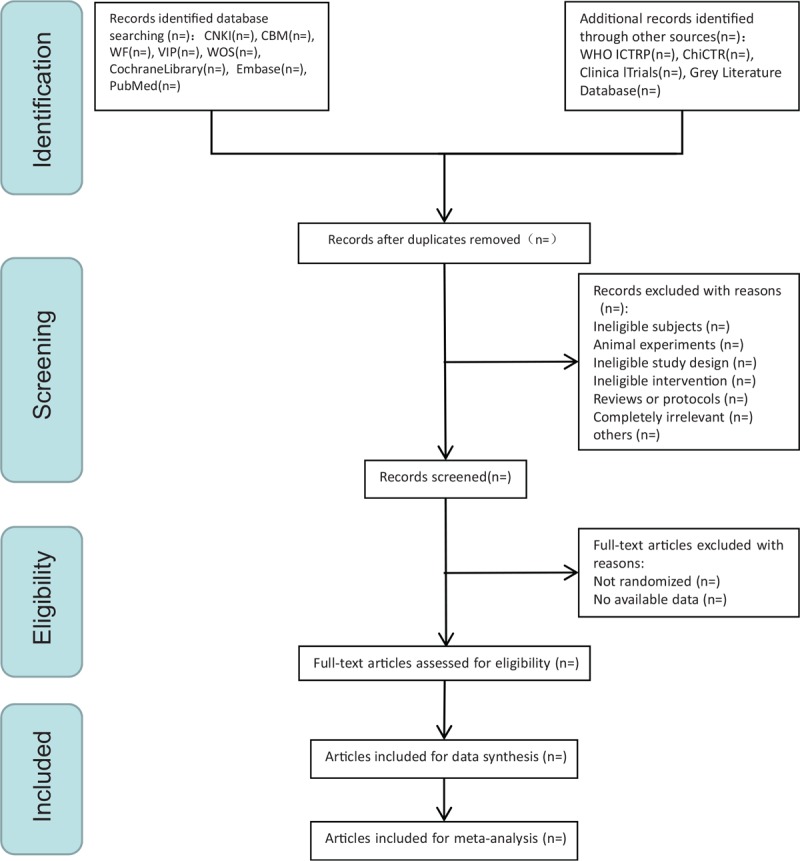
The Preferred Reporting Items for Systematic Reviews and Meta-analyses Protocols flow chart of selection process.

All reviewers will have a professional training about background, purpose, and process of the review. In the literature collection, the title and abstract of the literature will be 1st read to eliminate duplicate literature and the eligible studies searched will be uploaded to a database set up through NoteExpress. Two review authors will select and record independently through screening the titles, abstracts, and key words. Any disagreement about the inclusion of the studies will be resolved through discussion between the 2 review authors. If the discussion cannot reach an agreement, the arbiter will make a final decision of the study selection. If authors are similar or incidence data are extracted from the same database, the study period will be assessed. Details of the selection procedure for studies are shown in a PRISMA-P flow chart (Fig. 1)

#### 2.5.2 Data extraction and management

Data extraction will be also conducted by 2 researchers independently through a standardized eligibility form. In case of disagreement, a 3rd party (the arbiter) shall be consulted to assist judgment, and the missing information shall be supplemented by contacting the author. The general information of the selected articles will be extracted, including 1st author, year of publication, country, study design, sample size, detailed intervention, control treatment, duration of disease, duration of follow-up, and the like. When the data of articles are insufficient or ambiguous, one of the authors will in contact with the original author to request detailed about the research by e-mail or telephone or estimate the data.

#### 2.5.3 Assessment of risk bias

Two review authors will measure the risk of bias of the included studies with Cochrane Handbook V.5.3.0 independently, which includes the following 7 items: sequence generation, blinding of participants, blinding of outcome assessors, allocation concealment, incomplete outcome data, selective outcome reporting, and other sources of bias. It will be ranked risk level within categorized as low risk of bias, unclear risk of bias, and high risk of bias. In case of disagreement, the arbiter shall be consulted to assist judgment.

#### 2.5.4 Measures of treatment effect

The dichotomous data will be analyzed by relative risk (RR) ratio with 95% confidence intervals (CIs), and the mean difference (MD) or standard MD (SMD) with 95% CIs will be used to estimate the continuous data.

#### 2.5.5 Management of missing data

Where possible, we will analyze the data according to the intention-to-treat. If there is missing or incomplete data, we will contact the original investigator to verify the study characteristics and obtain missing numerical result data. If the missing data are not available, then this analysis will depend on the data available.

#### 2.5.6 Assessment of heterogeneity

According to the Cochrane Handbook, we will choose the *I*^2^ statistic to measure heterogeneity among the studies in every analysis. When *P* > .1, *I*^2^ < 50%, it is considered that there is no heterogeneity between the experiment, and the fixed effects model will be used for statistics, otherwise, the random effects model is adopted to analyze.

#### 2.5.7 Assessment of reporting biases

If the number of included studies is >10, we will use funnel plots to measure publication bias. If funnel chart is evenly distributed, it indicates no reporting bias, and vice versa.

#### 2.5.8 Data synthesis

The data will be analyzed and synthesized through Review Manager 5.3 software which from Cochrane Collaboration will be employed to compute the data synthesis. The fixed effects model (*I*^2^ < 50%) or random effects model (*I*^2^ ≥ 50%) will be selected. All data will be analyzed with 95% CIs. The dichotomous data will be analyzed by RR, and the continuous data will be analyzed by MD or SMD.

#### 2.5.9 Subgroup analysis

If we find substantial heterogeneity, subgroup analysis will be implemented according to acupuncture types, outcome measures, and the like.

#### 2.5.10 Sensitivity analysis

We will carry out sensitivity analysis to identify the quality and robustness of the results in the review. The principal criteria include methodological quality, sample size, and analysis issue (such as missing data's efficacy). The meta-analysis will be operated repeatedly.

#### 2.5.11 Grading the quality of evidence

The reviewers will use the GRADE rating standards.^[22]^ The GRADE system will be used to GRADE the obtained outcome indicators from 5 items of research limitations, inconsistency, indirectness, inaccuracy, and publication bias. In the case of the RCTs, the GRADE classifies the evidence of the outcome indicators evaluated by the system, and all the outcome indicators are graded by quality through the GRADE rating standards. Then, evidence quality will be rated “high,” “moderate,” “low,” or “very low” according to the GRADE rating standards. The quality of the evidence is high, indicating that future research is unlikely to change existing evidence; “moderate” indicates that future research may have an important impact on existing evidence, and may change the evaluation results; Being low-level indicates that future research is likely to have a significant impact on existing evidence and may change the evaluation results; “very low” indicating that all existing evidence is highly uncertain.

## 3 Publication plan

The systematic review will be published in peer-reviewed journals in both electronic and print versions.

## 4 Discussion

The PCS is used to describe a collection of symptoms experienced by patients following cholecystectomy, many of which may not be attributed to disorder of biliary system.^[23]^

In China, acupuncture is the most common therapeutic option for diseases with pain. It is proved by clinical systematic reviews^[24–26]^ that acupuncture reduces the symptom of PCS. Acupuncture also has good effects on nausea, vomiting, and gastrointestinal function illustrated in many SRs.^[27–30]^

It's a 1st SR about acupuncture treatment for PCS, we expect that this systematic review will provide clinical evidence for the efficacy and safety of current acupuncture treatment of PCS, provide medical staffs with more useful information, and provide patients with better advises. There may be some limitations in this SR, including different types of acupuncture, language limitations, lack of research, and the like, which may lead to substantial heterogeneity.

## Author contributions

**Conceptualization:** Zihan Yin, Ying Cheng, Fan-rong Liang.

**Data curation:** Qiwei Xiao, Han Yang, Yanan Fu.

**Formal analysis:** Qiwei Xiao, Guixing Xu, Han Yang.

**Funding acquisition:** Fan-rong Liang.

**Methodology:** Guixing Xu.

**Project administration:** Fan-rong Liang.

**Supervision:** Jun Zhou, Jiao Chen, Ling Zhao.

**Writing – original draft:** Zihan Yin, Ying Cheng.

**Writing – review & editing:** Zihan Yin, Ying Cheng.

Zihan Yin orcid: 0000-0001-8741-1205.
